# Evolution of Cooperation on Spatial Network with Limited Resource

**DOI:** 10.1371/journal.pone.0136295

**Published:** 2015-08-27

**Authors:** Yang Wang, Binghong Wang

**Affiliations:** 1 Department of Modern Physics, University of Science and Technology of China, Hefei, Anhui, 230026, P. R. China; 2 College of Physics and Electronic Information Engineering, Wenzhou University, Wenzhou, Zhejiang, 325035, P. R. China; 3 School of Science, Southwest University of Science and Technology, Mianyang, Sichuan, 621010, P. R. China; Lanzhou University, CHINA

## Abstract

Considering the external resource offered by environment is limited, here, we will explore the cooperation on spatial networks with limited resource. The individual distributes the limited resource according to the payoffs acquired in games, and one with resource amounts is lower than critical survival resource level will be replaced by the offspring of its neighbors. We find that, for larger temptation to defect, the number of the dead decreases with the resource amount. However the cooperation behavior is interesting, the lower total resource and intermediate temptation to defect can greatly promote the cooperation on square lattice. Our result reveals that the limited resource contributes most to the cooperation.

## Introduction

In the past decades, the social dilemma games have been widely investigated [1–3]. The most typical model is the prisoner’s dilemma game(PDG) [4–9]. In the original PDG, individuals can choose cooperation or defection. Both of them can receive payoff *R* upon mutual cooperation and *P* upon mutual defection. If one chooses to defect while its opponent cooperates, the cooperator receives *S* and the defector gets *T*. In the PDG, the ranking of the four payoff values is *T* > *R* > *P* > *S* and 2*R* > *T* + *S*. There exist two essential features for these game models. Firstly, cooperation strategy maximizes the highest collective payoffs. Secondly, the transformation of a player from the cooperator to defector can increase its own payoff [10–15]. Hence, a rational player may choose defection strategy even the cooperation can brings the highest collective payoff, which leads to the distinction of cooperation. But cooperation can spread in the structured population by forming the clusters to resist the invasion of defection. Meanwhile, various mechanisms and factors which can affect the cooperation on networks have been investigated, including inhomogeneous activity [16, 17], kin selection [10, 18], migration [19–21], social diversity [22–24], reputation [25], memory [26], payoff sharing [27], punishment [28–31] and reward [32, 33]. Another evolution model is Moran process on networks [34, 35], it contains two updating rules: Birth-Death Update and Death-Birth Update, respectively. For the former, the reproduction probability of an individual is proportional to its fitness, and then a randomly selected neighbor is replaced. While for the latter, the death operation happens firstly, and the site of the dead individual is replaced by the neighbors’ offspring according to their fitness. The traditional setup of individual’s death and other activities are mainly based on the consideration of that individuals need a lowest payoff to survive, but it neglects the effect of environment [36, 37].

In nature, the environment condition impels the resource distributing to each individual is limited. Hence, the most profitable strategy is in a minority. It has been demonstrated that cooperation and competition patterns can arise in these games [38–40]. In addition, the complete cooperator and defector are rare, and more realistic fact is the strategy of the individual is continuous. Base on these considerations, we introduce a limited resource Death-Birth Update model into spatial games. At each update, individuals distribute the limited system resource according to their game payoffs. Then the one whose resource amount is lower than critical survival value will be replaced by offspring of its neighbor, and the selection probability is proportional to resource. Due to the continuous strategy, the payoff of the individual is the expected value acquired from its direct neighbors by playing the prisoner’s dilemma games. We find that the intermediate resource can result in minimal number of dead individuals and highest cooperation level. Additionally, for larger resource, the intermediate temptation to defect is greastest contribution to the cooperation. While from the aspect of biodiversity, enough sufficient resource and appropriate temptation are more helpful to the existence of individuals with various strategies.

The paper is organized as follows: Section 2 is the detailed description of the model. In Section 3, we present the simulation results. The Section 4 is the discussion.

## Model

We apply the square lattice with periodic boundary condition to simulate the ecosystems. Initially, each individual is assigned a characteristic parameter *p*(For convenience, we denote characteristic parameter as *CP*) as “gene” to quantify the tendency of individual to cooperate and *p* will be the strategy of individual to participate in games. Environment parameter *c* denotes the total investment of surrounding to each individual. For simplicity, we consider the environment consumption is same for each individual, and we set the consumption to be 1. Then, the total consumption and investment for the system are *L* × *L* and *G*_*sys*_ = *cL* × *L*, respectively.

A full Monte Carlo step consists three steps: calculation of payoff, distribution of resource and Dead-Birth Update.

- Calculation of payoff. An individual with characteristic parameter *p*_*i*_ interacts with a *p*_*j*_ neighbor can acquire payoff *P*_*ij*_:
  $$P_{ij}=p_{i}p_{j}R+\left(1-p_{i}\right)p_{j}T+p_{i}\left(1-p_{j}\right)S+\left(1-p_{i}\right)\left(1-p_{j}\right)P,$$ (1)
  where *p*_*i*_ is the strategy(characteristic parameter) of individual *i* and *p*_*j*_ is the strategy of *i*’s neighbour *j*. For simplicity but without loss of generality, the payoff matrix for the PDG is rescaled as *R* = 1, *S* = 0, *T* = *b*, *P* = 0, where *b* is temptation to defect, meanwhile it also can be considered as the characterization of competitive intensity. Then *P*_*ij*_ = *p*_*i*_ *p*_*j*_ + (1 − *p*_*i*_)*p*_*j*_ *b*. The payoff *P*_*i*_ = ∑_*j*_ *P*_*ij*_ of individual *i* is the total payoffs acquired from its four neighbors and the total payoff of system is *P*_*sys*_ = ∑_*i*_ *P*_*i*_.
- Distribution of resource. The distribution of resource is based on the individuals’ payoffs, the resource distributed into individual *i* is proportional to its payoff:
  $$G_{i}=G_{sys}P_{i}/P_{sys}.$$ (2)
  After subtracting the consumption, the eventually resource for *i* is *H*_*i*_ = *G*_*i*_ − 1. Eq 2 indicates that the winners in the games can gain the higher payoffs.
- Death-Birth Update. It is considered that the individual who has negative resource *H*_*i*_ does not adapt to environment and will be replaced by the offspring of its neighbor, which is selected with probability proportional to their resources. If all of its neighbors are eliminated, the characteristic parameter of vacant site is randomly selected. Repeat process i), ii) and iii), the system will reach the stable state. For all simulations, we set the network size *N* = 100 × 100.

## Results

**Fig 1:**
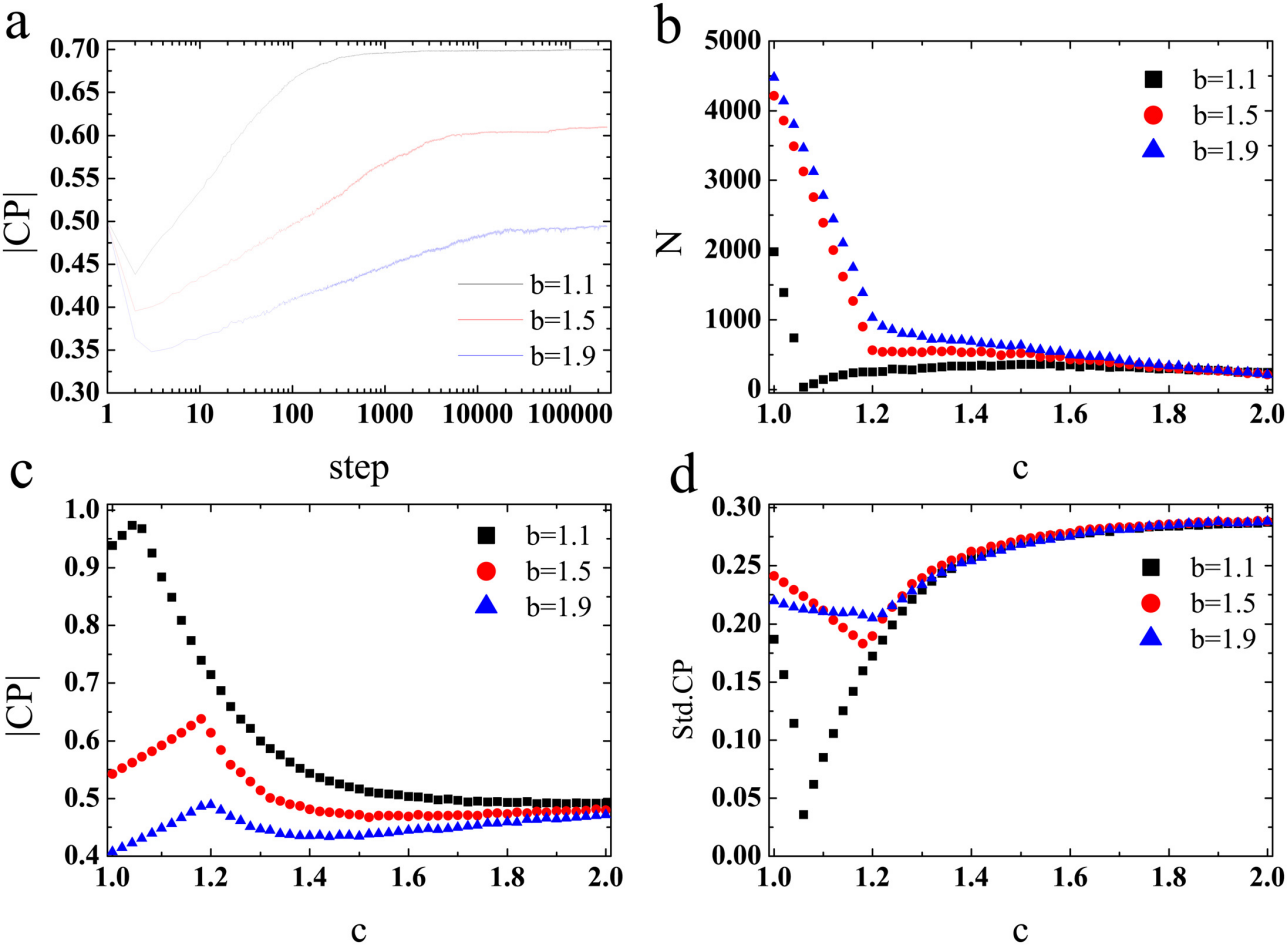
The cooperation on square lattice with limited resource. (a) Time evolution of average characteristic parameter *CP*. (b) The number of dead individuals as a function of resource rate *c* for different temptation to defection *b*. (c) The average characteristic parameter as a function of resource rate *c* for different temptation to defection *b*. (d) The standard deviation of *CP* for the system a function of resource rate *c* for different *b*. Each data point is an average of 100 independent realizations.

Fig 1(a) shows the evolution of average characteristic parameter(*CP*) for different *b*. Due to the advantage of lower *CP*, we can find the rapid declines of average *CP* in the most early stages of the evolutionary process. While the network reciprocity can protect the individuals with higher *CP* to resist the invasion of individuals with lower *CP*. Then, the average *CP* begins to increase in the subsequent evolution. For the typical prisoner’s dilemma games on spatial networks, cooperation is promoted by network reciprocity. For in cooperative clusters, cooperators have higher payoffs than their defection neighbors in competition domain. As time going on, due to advantage of cooperation, defectors gradually begin to adopt cooperation strategy. Again, in our model, individuals in higher *CP* clusters can obtain enough resources to survive. While the death of higher *CP* individuals who have not formed clusters gives rises to drop of payoffs and resources of lower *CP* individuals. Then, the sites occupied by lower *CP* individuals are replaced by individuals with higher *CP*.

Fig 1(b) shows the number of dead individuals as a function of resource rate *c* for different temptation to defect *b*. We find that, for strict environment, the larger *b* renders the more individuals be replaced by the offspring of their neighbors. For large *b*, the differences of payoffs between the individuals with lower *CP* and higher *CP* are also large, which makes the individuals with lower *CP* can gain more resources. Then, most of individuals with higher *CP* will be replaced by ones with lower *CP*. With the increasing of resource rate *c*, the number of dead individuals begins to decrease rapidly. Given the parameter *b*, the effect of environment selection is weak for large *c*, and most of the individuals can obtain a resource that larger than consumption. Interestingly, we discover that, for intermediate *c* and small *b*, the number of dead individuals reachs the minimum value. Intuitively, if the environment is advantageous, then the selection effect is weak. While the Fig 1(b) reveals that, for small temptation to defect *b*, the intermediate environment is the greatest contribution to the survival of individuals.

Fig 1(c) shows the average characteristic parameter(*CP*) of system as a function of resource rate *c* for different *b*. We find that, for *b* = 1.1 and strict environmental condition, average *CP* nearly equals to 1. This indicates that the strong environment selection can create the most healthy cooperation environment. Unlike the well-mixed population, the cooperators can survive by direct and indirect network reciprocity. In this point, our results indicate that, the cooperators can also survive by network reciprocity when facing the strict environmental condition. However, with the increasing of resource rate *c*, the average characteristic parameter begins to reduce. In other words, the healthy environment cannot induce healthy cooperation behavior. For the case of large *b*, there exists an optimal environment rate *c*(around 1.2), in which the average *CP* is the highest. Fig 1(d) shows the standard deviation of *CP* for the system as a function of resource rate *c* for different *b*, respectively. It can be seen that, for three different *b*, the environment rate *c* corresponding to minimum of standard deviation of *CP* and number of dead individuals is exactly the same. The intermediate environment rate against the diversity of *CP*.

To intuitively explain the cooperation on square lattice by incorporating the environmental effect, we divide the interaction of a pair of individuals into three categories: i) “parasites-hosts”. The individual with lower *CP*(parasites), but its neighbor has higher *CP*(hosts); ii) “mutual cooperation”. The individual with higher *CP* and its neighbor also with higher *CP*; iii) “mutual defection”. The individual with lower *CP* and its neighbor with lower *CP*; From Eq 1, it can be known that the payoff ranking is “parasites”, “mutual cooperation”, “mutual defection” and “hosts” if we array with the individuals’ profits from high to low, the ranking is also the order of individuals’ assorted resource and probability of survival.

**Fig 2:**
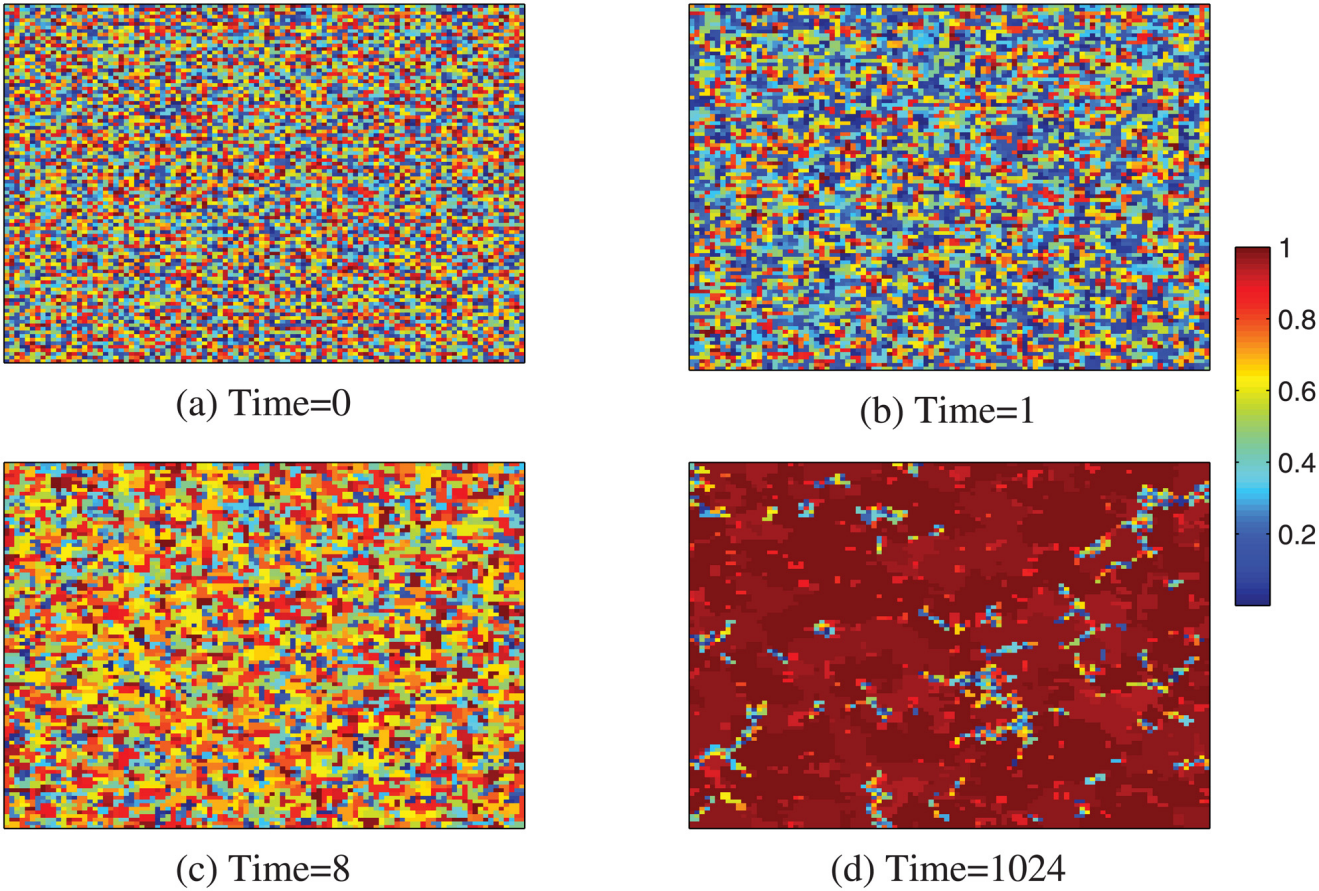
Evolution of cooperation from a random initial state with limited resource. The snapshots are taken at MCS = 0 (a), 1 (b), 8 (c) and 1024 (d). The system size *L* = 100 and *b* = 1.1, *c* = 1.01.

**Fig 3:**
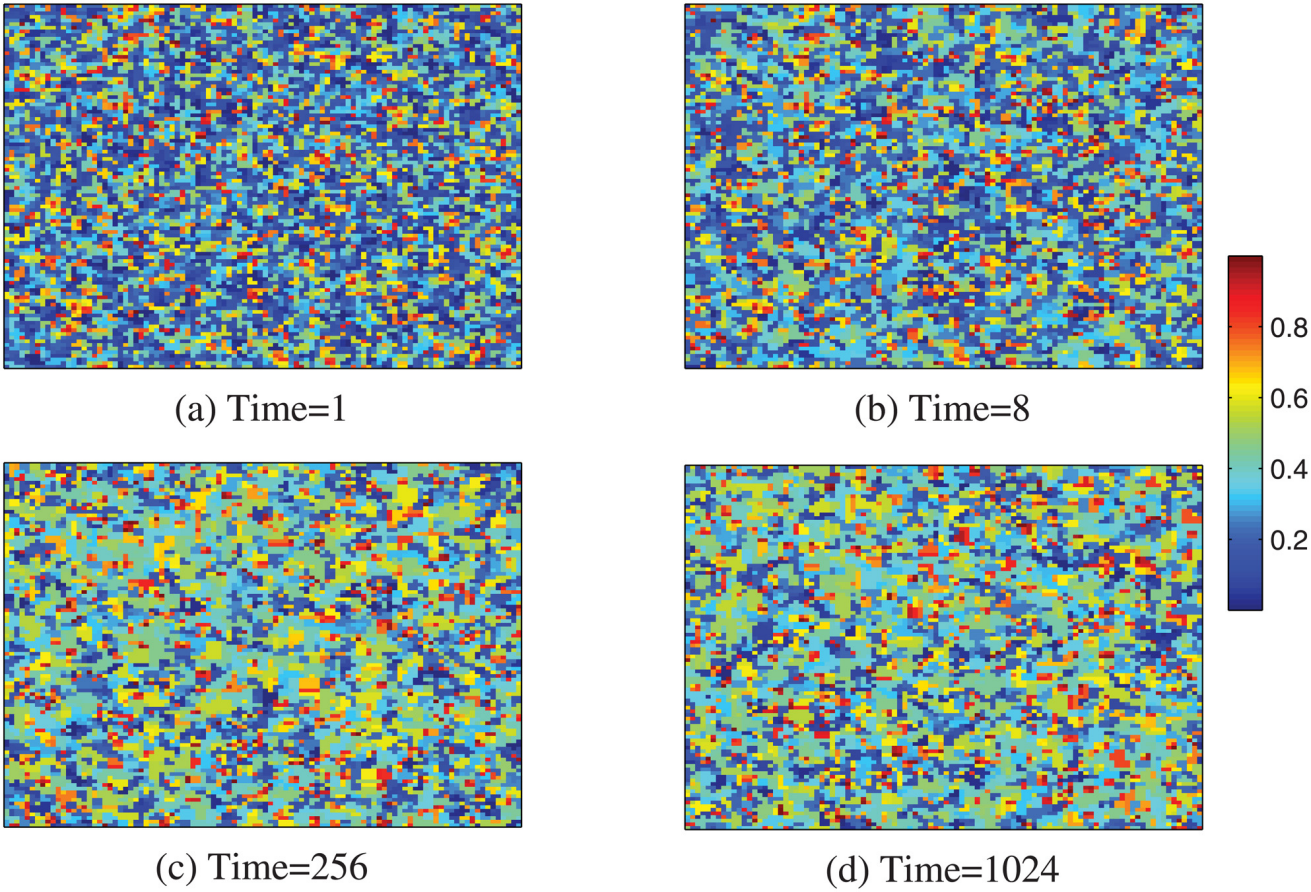
Evolution of cooperation from a random initial state with limited resource. The snapshots are taken at MCS = 1 (a), 8 (b), 256 (c) and 1024 (d). The system size *L* = 100 and *b* = 1.9, *c* = 1.01.

**Fig 4:**
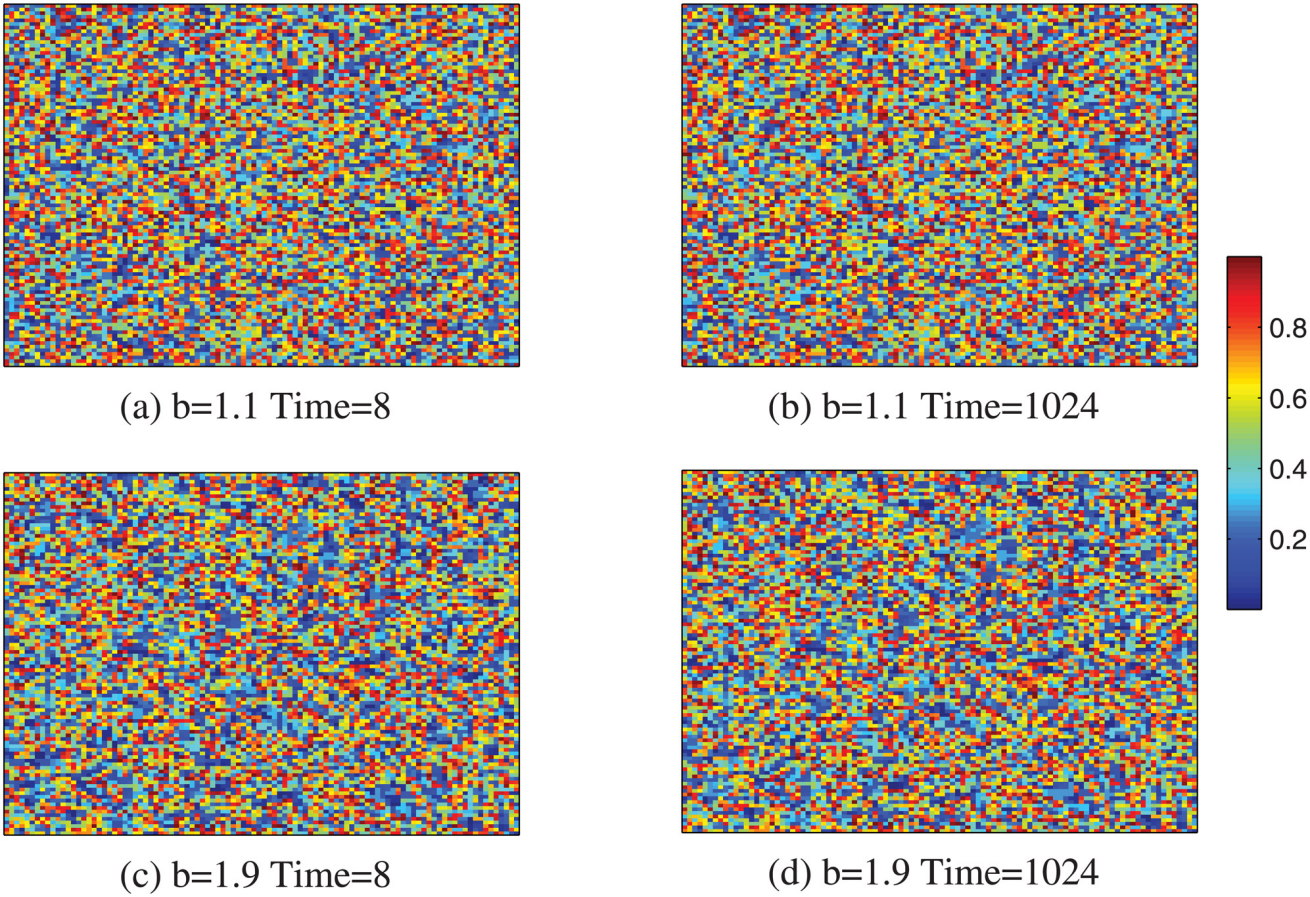
Evolution of cooperation from a random initial state with limited resource. The snapshots are taken at *b* = 1.1, *MCS* = 8 (a), *b* = 1.1, MCS = 1024 (b), *b* = 1.9, *MCS* = 8 (c) and *b* = 1.9, *MCS* = 1024 (d). The system size *L* = 100 and *c* = 1.9.

Figs 2–4 show the evolution of cooperation from a random initial state with limited resource for different temptation to defection. In Fig 2, we find that, for lower resource amount, at the beginning of MCS, the hosts and individuals in mutual defection are replaced rapidly by their neighbors. While the individuals in mutual cooperation can survive by network reciprocity. As the hosts and individuals with lower *CP* in mutual defection die out, and the empty sites are also replaced by individuals with higher *CP*, so lower resource and temptation to defection *b* can greatly promote the cooperation on lattice networks. While with the increasing of resource rate *c*, most individuals can survive in the system. Besides, the evolution of cooperation nearly holds unchanged, as shown in Fig 4. In this, the average *CP* also trends to 0.5, which is consistent with the result of Fig 1(b).

**Fig 5:**
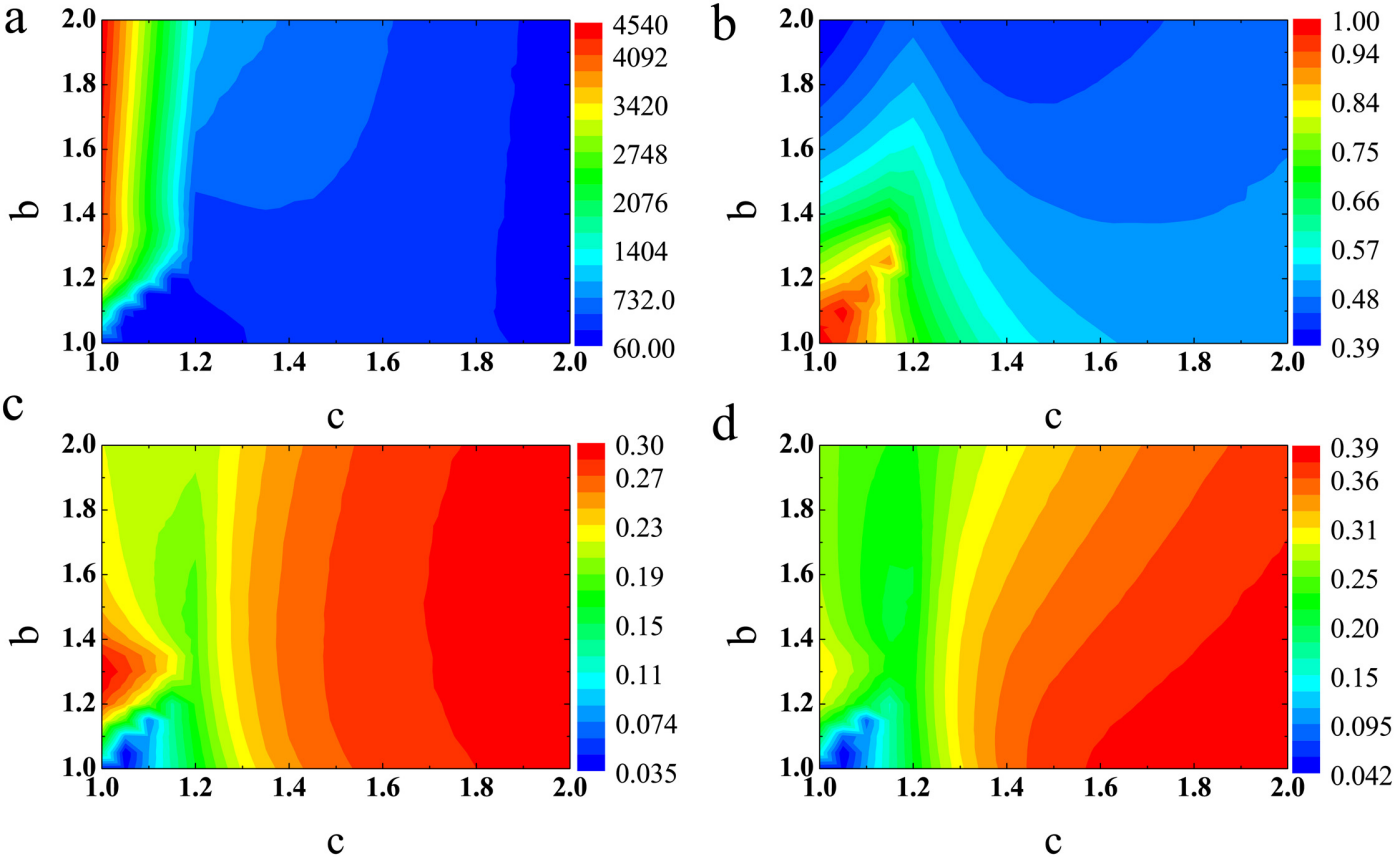
The cooperation on square lattice with limited resource. (a) The number of dead individuals on the *c* − *b* parameter plane. (b) The average characteristic parameter on the *c* − *b* parameter plane. (c) The standard deviation of *CP* on the *c* − *b* parameter plane. (d) The average characteristic parameter on the *c* − *b* parameter plane in a small parameters region(1.0 < *c* < 1.4,1.0 < *b* < 1.4). Each data point is an average of 100 independent realizations.

Finally, we explore the average number of dead individuals, average *CP* and standard deviation as functions of temptation to defect *b* and resource rate *c*, as shown in Fig 5. Fig 5(a) shows that, the number of dead individuals is mainly affected by the resource rate. It decreases rapidly with *c*, but slightly increases with *b*. Hence, the system with enough resource or more healthy cooperation environment may induce the total population to increase gradually. Fig 5(b) shows that, the system with lower temptation to defect *b* and *c* has most healthy cooperation environment. Fewer resources render the individuals with higher *CP* to form the clusters and further eliminate the invasion of lower *CP* individuals. While for the system with more enough resource, each individual can survive irrespective higher *CP* or lower *CP*. While Fig 5(c) shows that, the lower *b* and *c* are not contribution to the diversity of *CP*. To more clearly understand the effect of temptation to defect *b* and resource rate *c* on the average *CP*, we set 1 < *b* < 1.4 and 1 < *c* < 1.4. Interestingly, as *c* around 1.1, there exists an optimal average *CP* at middle temptation to defect *b*.

## Discussion

The former works related to the evolutionary games on spatial networks are mainly focused on how to promote the cooperation among selfish individuals. The recent works have also begun to concern the cascading failure [37], silence [36] and other behaviors in game dynamics. While these behaviors are induced by individuals’ payoffs. The resource offered by the environment is usually limited, and the resource distributed by the system can be affected by games. Hence, we introduce the limited resource induced cooperation on spatial networks. Individuals with negative resource will be replaced by their neighbors’ offspring. We find that, the lower resource amount and temptation to defect can greatly promote the cooperation and have more strategy diversity. But the average number of dead individuals is deduced with the increasing of resource amount. As a result, the limited resource amount is the greatest contribution to the cooperation.
